# Myomatous Erythrocytosis Syndrome: Case Report and Review of the Literature

**DOI:** 10.7759/cureus.6892

**Published:** 2020-02-05

**Authors:** Pooja Suresh, Sanaa Rizk

**Affiliations:** 1 Hematology and Medical Oncology, Abington Hospital – Jefferson Health, Philadelphia, USA; 2 Hematology, Thomas Jefferson University Hospital, Philadelphia, USA

**Keywords:** myomatous erythrocytosis syndrome, erythropoietin

## Abstract

Myomatous Erythrocytosis Syndrome is defined as erythrocytosis, myomatous uterus, and the return of normal hematologic values following surgical resection. The exact role of erythropoietin in disease pathogenesis is unknown. In this study we report the case of a 49 year old premenopausal woman who was found to have an enlarged heterogeneous mass arising from the uterus concerning for malignancy. Her RBC count was 5.75 T/L, hemoglobin was 17.6 g/dL and hematocrit was 54.3%. Pre-operative erythropoietin levels were 24.6 mIU/mL and JAK2 mutation was not detected. She underwent Total Abdominal Hysterectomy and Bilateral Salpingo-Oophorectomy. The pathology was consistent with a uterine leiomyoma. Laboratory evaluation performed eight weeks after surgery showed a RBC count of 4.5 T/L, hemoglobin of 13.6 g/dL, hematocrit of 40.5%. Post-operative erythropoietin level was 5.4 mIU/mL. The tissue showed diffuse moderate to strong cytoplasmic immunopositive for Erythropoietin. Erythropoietin plays an important role in this condition, however the exact mechanism is still under investigation. The theory of erythropoietin secreting tumor autonomously without negative feedback is the most credible so far. However, further studies with use of blood erythropoietin level, tissue erythropoietin detection using immune-stain and new molecular biology techniques need to be done and compared to uterine myoma patients with no erythrocytosis. Usually, no further treatment is required following surgical removal.

## Introduction

The triad of a myomatous uterus, erythrocytosis, and restoration of normal hematologic parameters following surgical resection such as hysterectomy or myomectomy is characteristic of a rare entity called Myomatous Erythrocytosis Syndrome. The first reports of these conditions were by Thomson and Marson in 1953 [1]. The exact pathogenesis of this disease is unknown. However, there have been published case reports since then examining various hypotheses, which we will discuss here. Erythrocytosis has been frequently seen in association with some malignancies such as renal cell carcinoma and cerebellar hemangioblastoma. However, all reported cases of this syndrome have been fortunately associated with benign leiomyomas. We will report a case of a premenopausal woman with erythrocytosis and enlarged uterine leiomyoma with a detailed literature review.

## Case presentation

**Figure 1:**
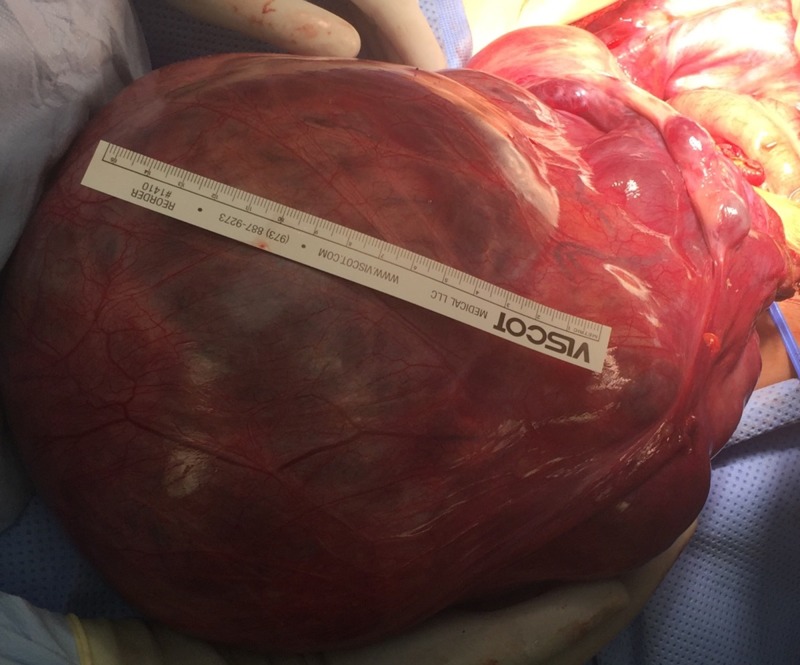
Post-operative gross specimen- Large abdominopelvic mass originating from the uterus measuring approximately 31 cm in its largest diameter and weighing 5.54 kg.

A 49 year old Caucasian, pre-menopausal woman, gravida 1, para 1, was referred to our office for evaluation of erythrocytosis. She initially presented to her primary care physician with the inability to lose residual abdominal fat after intentionally losing 50 pounds over the past 1-2 years. A CT abdomen, pelvis, and subsequent MRI revealed a large heterogeneous solid mass with internal vascularity originating from pelvis and extending into abdomen, apparently originating from uterus, measuring 13.3 x 24.1 x 26.3 cm with neovascularization. There was also mild bilateral hydronephrosis, likely due to extrinsic mass effect on the ureters. She was subsequently referred to the Department of Gynecology for further evaluation. Initial laboratory tests revealed a red blood cell count of 5.75 T/L (normal 3.7-5.2 T/L), an elevated hemoglobin level of 17.6 g/dL (normal range 11.7-15.5 g/dL), an elevated hematocrit of 54.3% (normal 35-45%), a normal white blood cell count of 5.7 K/uL, and a normal platelet count of 184 K/uL. We evaluated her prior to surgical resection of her pelvic mass. She denied any symptoms of hyperviscosity such as headache or blurry vision. She denied any erythromelalgia. She had no significant medical history and denied any prior history of thrombosis or abnormal blood work in the past. She was not a smoker and did not live in high altitude. She denied any history of lung disease. She was not aware of any snoring habits during her sleep or hyper somnolence during daytime. She denied any family history of red blood cell elevation or other hematologic disorders. She denied any medication use as well as any over the counter drug or herbal remedies. Her menstrual cycle was regular, occurring every 28 days, with moderate bleeding, requiring changing pads every 2-3 hours for the first couple of days, followed by a lighter bleed for extra 3 days or so. Her physical exam was benign except for an enlarged, irregular, palpable mass involving almost the entire abdominal cavity without any associated lymphadenopathy. Laboratory evaluation in our office showed an erythropoietin level of 24.6 mIU/mL (normal 2.6-18.5 mIU/mL). JAK2 mutations (V617F and exons 12 and 13) were not detected. Arterial embolization of uterine and left ovarian arteries was performed prior to surgery to minimize blood loss. She then underwent an exploratory laparotomy which revealed a large uterine mass extending to the xiphoid process which was resected. The mass measured 31 x 22 x 14 cm and weighed 5.54 kg (Figure 1 ).

A Total Abdominal Hysterectomy and Bilateral Salpingo-Oophorectomy were also performed. There was minimal intra-operative blood loss recorded. Pathology was consistent with leiomyoma with patchy areas of intratumoral hemorrhage and vascular congestion. There was no evidence of malignancy. On postoperative day one, her hemoglobin had normalized to 12.6. Her postoperative course was uncomplicated. Her blood work at eight weeks post-op showed a red blood count of 4.5 T/L and her hemoglobin at 13.6 g/dL with a hematocrit of 40.5%. The repeat erythropoietin level had decreased to 5.4 mIU/mL.

**Figure 2:**
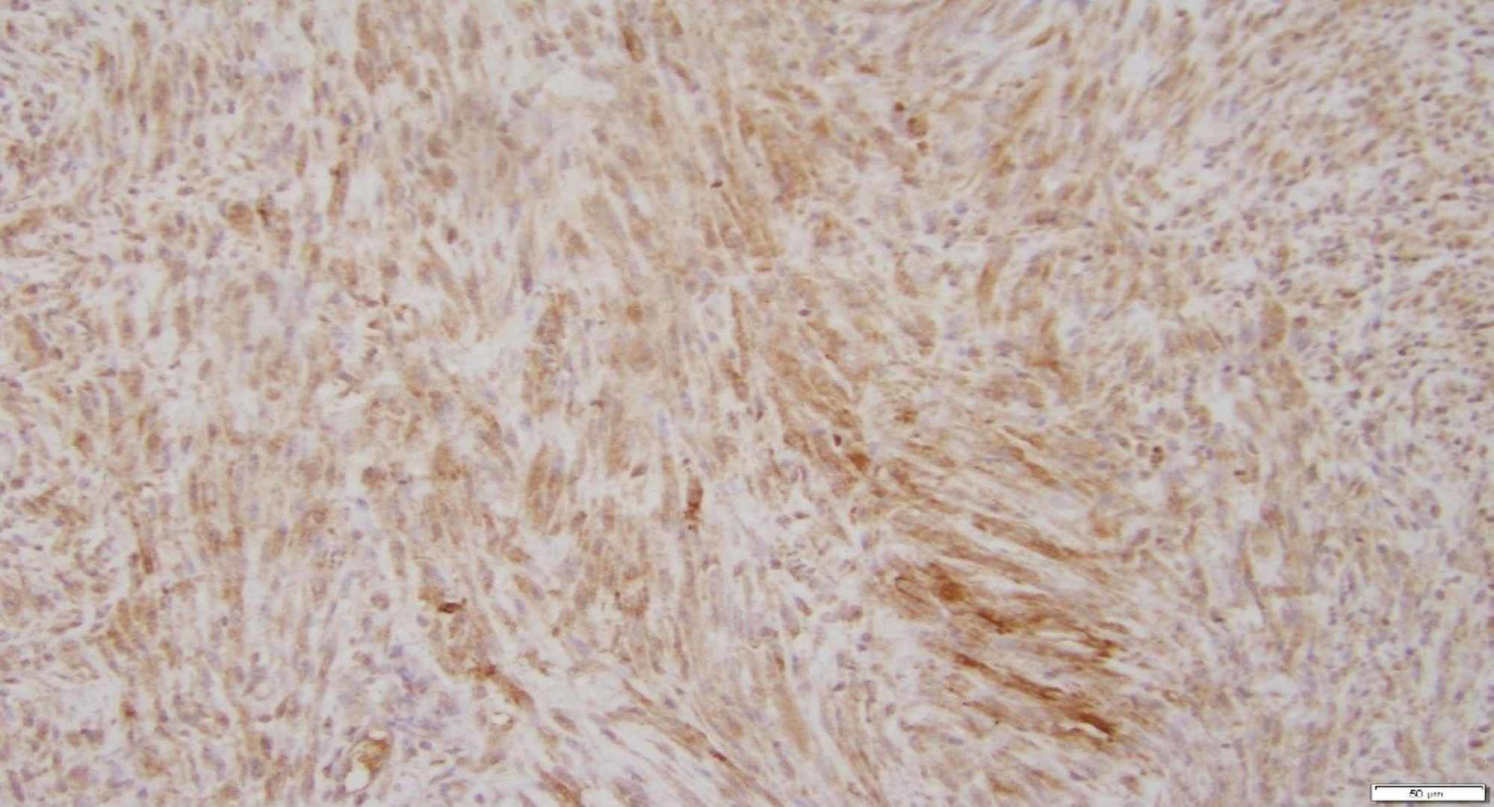
A representative section of the large leiomyoma shows diffuse moderate to strong cytoplasmic immunopositive for Erythropoietin (200x EPO IOHC).

To prove that erythropoietin was produced from the uterine leiomyoma, we performed an immunostaining for erythropoietin on this tissue using a rabbit polyclonal antibody directed against amino acids 28-189 of human erythropoietin (Santa Cruz Biotechnology, Inc). The cytoplasm of the leiomyoma cells stained moderate to strongly positive for erythropoietin (Figure 2 ).

## Discussion

The most common presentation for uterine myomas is increased menstrual bleeding, leading to anemia most of the times. However, in rare cases, despite menorrhagia, the hemoglobin may be disproportionally normal or elevated. Thomson and Marson were the first to report erythrocytosis secondary to uterine myoma in 1953 [1]. Since then, there have been around 50 cases reported in the world literature. The actual incidence of this syndrome is not well established among the English literature, likely due to concurrent heavy vaginal bleed causing falsely normal hemoglobin values. Many hypotheses regarding pathophysiology of myomatous erythrocytosis syndrome has been suggested.

One of the theories presented by Horwitz and McKelway in 1955 was the presence of arteriovenous shunt, which would lead to the presence of deoxygenated blood flow in the arterial system, and marrow stimulation to increase RBC production as a result [2,3]. However, this theory was rejected since the microscopic appearance of myomas with erythrocytosis was similar to myomas without erythrocytosis. There has also been lot of case reports of patients with uterine AV fistulas without concurrent erythrocytosis.

Menzies et al described compression of these uterine masses causing disruption of urinary flow leading to increased renal parenchyma pressure and erythropoietin stimulation, or even compression of renal vessels leading to renal hypoperfusion and erythropoietin secretion [4].

Paranjothy et al also attributed the increased size of uterine myomas to causing pressure on the diaphragm leading to hypoxia and subsequent increase in erythropoietin production [5]. This theory, however, was aborted since many patients with this condition did not show any signs of hypoxia or abnormal pulmonary function tests [2].

Production of erythropoietin happens mainly in the renal cortex and is involved in survival, growth, and differentiation of progenitor cells of the erythroid lineage. In rare scenarios, erythropoietin may be produced by some tumor cells, like renal cell carcinoma, hepatocellular carcinoma, and cerebellar hemangioblastoma. The first time the theory of uterine myomas producing excess erythropoietin was proposed in 1955 [3]. In 1968, Hertko et al. was the first one to discuss increased radioactive iron incorporation in a hypoxic polycythemic mouse [6,7]. Wrigley et al. later confirmed this using a 2-dose level radioactive iron assay in 1971 [8]. Since then, many have shown increased erythropoietin activity in uterine myoma tissue. Among them, Yoshida et al. demonstrated positive immune-staining for erythropoietin in the cytoplasm of leiomyoma cells [9]. Erythropoietin produced directly by uterine myoma tissue has also been demonstrated by Kohama et al. who used reverse-transcriptase polymerase chain reaction and Southern blot method to detect erythropoietin mRNA in myomatous tissue [3,10]. There is some in-vivo experimental data to suggest that estrogen plays a role in stimulating the erythropoietin/erythropoietin-receptor signaling pathway causing increased angiogenesis, which is important in the development of this syndrome [11]. Literature review showed associated cases of erythrocytosis with leiomyoma of the esophagus and cutaneous leiomyoma, bringing up the theory of myoma cells itself being responsible for inappropriate erythropoietin secretion, regardless of its location.

Apart from confirming erythropoietin production by myomatous tissue, Fabrizio Pollio and colleagues found a strong Epo-R expression in myoma endothelial cells, leading to the theory that erythropoietin production from uterine myoma not only contributes to erythrocytosis, but also stimulates proliferation and differentiation of myoblasts through autocrine and paracrine mechanisms leading to the large myomatous size almost always seen in this condition [12]. Similarly, in our case, we were able to detect elevated erythropoietin secretion in the myomatous tissue with elevated erythropoietin level in the blood that normalized after surgical resection of the leiomyoma.

In our case, it could be direct erythropoietin secretion from the tumor as proven by positive immunostaining performed on the tissue. The actual mechanism of disease pathogenesis is still unknown. It is most likely multifactorial, with the theory of the leiomyoma secreting erythropoietin autonomously without negative feedback being the most credible so far. However, further studies with use of blood erythropoietin levels and tissue erythropoietin detection using immune-stain and new molecular biology techniques need to be done and compared to uterine myomas without erythrocytosis. This would help shed more light on this syndrome.

In the setting of elevated hemoglobin and hematocrit, it is imperative to rule out Polycythemia Vera, a myeloproliferative neoplasm resulting in uncontrolled erythropoiesis of the bone marrow. Detection of JAK2 mutation and a low erythropoietin level are diagnostic of Polycythemia Vera. This can lead to serious, life-threatening thrombosis. Therefore, early recognition is important as treatment for Polycythemia Vera is different than secondary erythrocytosis due to a myomatous uterus, which resolves after surgical removal of the uterus.

Based on the frequency of uterine fibroids in women, it is unclear as to why there are not more cases Myomatous Erythrocytosis Syndrome. More research in the future needs to be done to further understand this rare condition.

## Conclusions

Myomatous Erythrocytosis Syndrome is a rare presentation of uterine fibroids, since most people present with anemia. It is important to recognize the association between erythrocytosis and fibroids to minimize unnecessary testing. The exact role of erythropoietin in disease pathogenesis is unknown. It is most likely multifactorial, with the theory of erythropoietin secreting tumor autonomously without negative feedback being the most credible so far. However, further studies with use of blood erythropoietin level, tissue erythropoietin detection using immune-stain and new molecular biology techniques need to be done and compared to uterine myoma patients with no erythrocytosis.
